# Can Mismatch Negativity Be Used as an Indicator to Predict Central Auditory Deficits in Individuals with Normal Hearing?

**DOI:** 10.3390/audiolres15020043

**Published:** 2025-04-16

**Authors:** Lichun Zhang, David Mißler, Karsten Ehrt, Wilma Großmann, Robert Mlynski, Florian Herrmann Schmidt

**Affiliations:** Department of Otorhinolaryngology, Head and Neck Surgery, ‘Otto Körner’, Rostock University Medical Center, Doberaner Straße 137–139, D-18507 Rostock, Germany; david.missler@uni-rostock.de (D.M.); karsten.ehrt@med.uni-rostock.de (K.E.); wilma.grossmann@med.uni-rostock.de (W.G.); robert.mlynski@med.uni-rostock.de (R.M.); florian.schmidt@med.uni-rostock.de (F.H.S.)

**Keywords:** cochlear synaptopathy, mismatch negativity, age related hearing loss, presbyacusis

## Abstract

**Background/Objectives**: In the early stage of presbycusis, patients experience reduced speech perception in noisy environments despite normal audiometry, normally known as hidden hearing loss. Diagnostic indicators like the reduced amplitude of ABR wave I, elevated extended high-frequency threshold (EHT), and decreased middle ear muscle reflex (MEMR) amplitude aim to identify biomarkers of peripheral auditory pathology but remain inconsistent. Mismatch negativity (MMN) is a cortical auditory evoked potential generated when the brain detects sound changes. This study aimed to assess MMN as a diagnostic tool for hidden hearing loss in adults. **Methods**: Seventy-three subjects with normal hearing underwent an extended pure-tone audiogram examination ranging from 0.125 to 16 kHz and a subsequent MMN assessment with two different paradigms: a speech (ba/da) and a tone (1/2 kHz) paradigm. The MMN’s amplitude and latency were measured and analyzed. **Results**: The outcome shows a significant age-related effect on MMN amplitude in the speech condition (χ² = 13.0, *p* = 0.002). Specifically, the MMN amplitude in the 25–30-year-old group was significantly smaller than in the 20–25-year-old group (*p* = 0.0015, Cohen’s d = 0.63). Additionally, no further effects of age were observed on the cortical potentials examined. Also, neither tone nor speech paradigms showed a significant influence of EHT on the amplitude or latency of either MMN or P300. **Conclusions**: The application of MMN as an electrophysiological tool to diagnose hidden hearing loss in normal hearing adults has limitations. However, in contrast to MMN responses to tonal stimuli, the present study reveals that MMN amplitude obtained with speech stimuli may indicate early signs of central auditory deficits.

## 1. Introduction

Age-related hearing loss (ARHL), also known as presbycusis, is the most common sensory deficit affecting adults, which increases with age and can reach a prevalence of 84.3% among people over 80 years old [1,2]. The estimated prevalence is that more than 500 million individuals over 60 will be affected by 2025 [3]. Typical patients exhibit progressive, irreversible sensorineural hearing loss that deteriorates with age, mainly involving the high-frequency range and gradually spreading to the low-frequency range [4]. ARHL affects not only the cochlear structures but also the central nervous system, leading to a reduced ability to process auditory information [5].

Recent studies predominantly focused on the early stage of ARHL and its potential mechanisms [6,7,8]. At this stage, patients mainly experience difficulties in speech perception, especially in challenging listening situations like environments with background noise. A typical example is the “cocktail party phenomenon”. This issue often goes unnoticed in routine clinical practice because standard hearing tests fail to detect changes in hearing thresholds in such cases. Consequently, Schaette and McAlpine (2011) referred to this condition as “hidden hearing loss” [9]. Animal studies suggest that cochlear synaptopathy may be the primary pathological mechanism underlying hidden hearing loss [10,11]. In these studies, a reduction in wave I amplitude has been proven to be a useful diagnostic marker [10,12,13,14]. However, in humans, ABR wave I is typically small and highly variable, limiting its diagnostic value for cochlear synaptopathy [15]. As a result, several alternative diagnostic metrics have been developed and studied, such as envelop-following response (EFR), acoustic middle ear muscle reflex (MEMR), and ABR wave I curvature [15,16,17,18]. Additionally, Electrocochleography (ECochG) has been explored using the ratio of summation potential (SP) to action potential (AP), as well as the amplitude of cochlear microphonics (CM) [19,20,21,22]. Extended high-frequency threshold (EHT) measurements have also been investigated [16,18]. Among these metrics, EHT is considered a highly sensitive biomarker for detecting early changes in the peripheral auditory pathway and may serve as an indirect indicator of cochlear synaptopathy [16,18]. Animal studies have shown that cochlear synapses in the mid and apical regions are more vulnerable than the outer hair cells (OHCs) in the far base [23,24]. Therefore, an increase in EHT may indicate cochlear synaptopathy occurring at least in the mid and/or apical region. Additionally, EHT can easily be performed in a clinical setting and is both faster and easier to analyze compared to the aforementioned measurements [18]. Therefore, this study aims to use EHT as a marker of the earliest changes in the peripheral auditory pathway.

How does the central nervous system change at the early stage of ARHL? Pathological changes in the peripheral auditory pathway lead to reduced signal input to the central nervous system, causing compensatory downregulation of inhibitory processing. This mechanism is known as central gain. While central gain can restore perception of sounds in quiet environments, it cannot recover the processing of temporal details [25]. Previous studies proved that the consequences of central gain extend beyond merely increasing central responses and may also contribute to tinnitus and/or hyperacusis [26,27,28,29]. All this evidence highlights that sensory deficits, specifically pathological changes in the peripheral auditory pathway, have complex effects that extend beyond central gain alone [30]. Currently, there is no clear understanding of the mechanisms by which central gain influences target sound processing in background noise.

Mismatch negativity (MMN), the cognitive component of the auditory evoked brain response, is a late latency response that provides an objective and non-invasive electrophysiological measure of cortical potential that reflects the brain’s automatic detection of stimulus differences [31,32,33]. MMN represents the capability of auditory discrimination and provides reliable results in evaluating the central auditory system. MMN is recorded using an oddball paradigm, in which a deviant or rare stimulus is presented within a series of homogenous or standard stimuli [31,34]. This study aims to record MMN responses using different types of stimuli across various age groups to investigate how these responses change with age, especially in the early stage of ARHL. Additionally, it examines how potential peripheral cochlear synaptopathy affects central auditory processing by analyzing the correlation between MMN and EHT. This approach may offer more reliable and objective diagnostic metrics for hidden hearing loss in the future.

## 2. Materials and Methods

The local ethics committee approved this prospective study (A 2023-0010). Written information was provided to the participants of the study group, and their consent was given. All personal data were anonymized and de-identified prior to the analysis.

### 2.1. Subjects

This prospective study included adults with normal hearing. Recruitment took place at the local university from January to June 2023 and involved students and staff members. All participants were young and healthy, without a medical history or regular medication use. In particular, none of the participants had a history of ear problems or reported subjective hearing loss. Additionally, we inquired about their history of noise exposure by asking them to recall significant experiences from their lives. Upon recruitment, each participant underwent a physical examination by an ENT consultant. After that, all participants underwent audiometric tests by qualified personnel in both ears using a clinically calibrated audiometer (AT900, Auritec GmbH, Hamburg, Germany). Pure-tone stimuli were presented at frequencies of 0.125 to 16 kHz. Frequencies from 0.125 to 8 kHz were presented by DT48 headphones, while extended high-frequency regions (10, 11.2, 12.5, 14, and 16 kHz) were presented by HDA200 circum-aural headphones (Sennheiser, Wedemark-Wennebostel, Germany). Otoscopy revealed that all participants had normal eardrums and ear canals. Participants were excluded from the study if their hearing threshold exceeded 30 dB HL at any conventional frequencies between 0.125 and 8 kHz or if their average hearing threshold at 0.25, 0.5, 1, 2, and 4 kHz exceeded 20 dB HL [35]. Furthermore, we excluded ears that did not exhibit a measurable MMN response in at least one of the two measurement paradigms.

### 2.2. Stimulation and Recording

Two MMN experiments were carried out: one based on a tone paradigm and another on a speech paradigm. In the tone paradigm, 100 ms tone pulses were used, featuring a 40 ms plateau and 30 ms onset and offset. The standard stimulus was a 1 kHz tone pulse. The deviant stimulus was a 2 kHz tone pulse. The stimuli and the measurement procedure are part of the standard repertoire of the ERA system. In the speech paradigm, the syllables b‘a’ and d‘a’ were used to trigger an MMN. The b‘a’ stimulus, used as the standard, had a duration of 150 ms at a sample rate of 30 kHz. Conversely, the deviant, represented by the d‘a’ stimulus, also operated at a sample rate of 30 kHz but with a duration of 40 ms. The probability of the deviant stimulus was 16%. All stimuli were presented monaurally at a level of 70 dB nHL for each ear in randomized order. The stimuli were presented through earphones calibrated for unshielded insert by a technician from Diatec Diagnostics GmbH, the company responsible for the regular clinical maintenance of the ERA system. The stimuli were presented in identical conditions using insert earphones (E-A-RTONE Insert Earphone, 3M, USA).

The electrophysiological measurements were performed using the Eclipse ERA-system (Interacoustics A/S, Middelfart, Denmark) at a sampling rate of 500 Hz. AgCl electrodes (Ambu BlueSensor N, Ambu GmbH, Bad Nauheim, Germany) were placed prefrontally on the midline sagittal plane of the skull (Fpz) and on the ipsilateral mastoids (TP9 or TP10), and the ground electrode was positioned on the central forehead. To ensure electrode contact, the skin area was prepared with alcohol and conductive gel, maintaining a contact impedance of less than 3 kΩ. During the recording session, participants lay on an examination bed in a sound-isolated room (GTA < 40). They were instructed to minimize blinking as much as possible, to fixate their eyes on a cross positioned on the opposite wall, and to keep their neck and shoulder muscles relaxed.

### 2.3. Data Processing and Analysis

The online analysis included digital filtering from 1 to 33 Hz. The oddball paradigm involved presenting a sequence of standard and deviant stimuli. On average, there were 336 trials for the standard stimulus and 64 trials for the deviant stimulus, corresponding to probabilities of 84% and 16%, respectively. The cortical responses were recorded within a recording window of −85 to 850 ms relative to stimulus onset. The recording session lasted approximately 20 min. All recorded data were imported into MATLAB (Version 2014a, Massachusetts: The MathWorks Inc., Natick, MA, USA) for additional offline processing. The samples of the raw data were excluded if they exceeded an artifact threshold of 30 µV to minimize eye artifacts. Afterward, the data were filtered using a second-order butter worth filter design, with a low-pass cutoff at 25 Hz and a high-pass cutoff at 1 Hz, ensuring zero-phase digital filtering. The baseline was adjusted by subtracting the mean EEG response before stimulus onset, sample by sample. The samples were averaged using an iterated weighted averaging procedure [36] with two iterations. This procedure provided weights for each epoch according to its estimated amount of noise contamination.

Our study investigates the differences in amplitudes and latencies of the cortical components MMN and P300. We established appropriate time windows for analyzing these potentials based on grand averages. These windows were both physiologically relevant and consistent with the latencies of both potentials in the literature. The time windows were set at 70 to 180 ms for MMN and 300 to 500 ms for P300.

**Figure 1:**
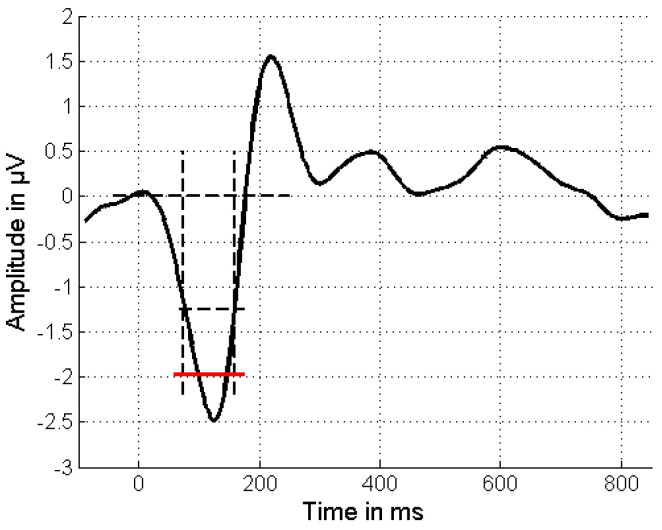
Measurement of the amplitude of the potentials. Long horizontal dashed line: baseline; short horizontal dashed line: 50% amplitude between peak and baseline; vertical dashed lines: time window of the critical duration, defined by the 50% amplitude values of the rising and the falling slope; red line: average amplitude across the critical time window.

To measure the amplitude of the potentials, the average amplitude was calculated within a time window that covered the critical duration of each potential. Within these windows, the peak of the potential was centered and the boundaries defined where the amplitude decreased to half its peak relative to both the peak and the baseline. This approach allowed us to represent the potential’s amplitude as the mean amplitude within this specified window (Figure 1).

Pearson correlation was performed on the electrophysiological measures, ears, age, and the mean EHT. To further investigate the effects of age and EHT, participants were divided into subgroups based on these factors for better comparison. For analyzing the impact of age, we used five-year increments, resulting in the following three groups: (1) 20–25 years old, (2) 25–30 years old, and (3) 30–35 years old. Similar, participants were grouped based on EHT into the following categories: (1) EHT < 0 dB HL, (2) 0 dB HL ≤ EHT ≤ 15 dB HL, and (3) EHT > 15 dB HL. The ‘Grand Average’, calculated as the arithmetic mean, was used to represent the overall EEG response across all measurements, reflecting total neural activity. The median was not used due to potential discontinuities in the temporal signal. However, for statistical analysis, non-parametric tests were employed, as the assumption of a normal distribution of the samples cannot be reliably verified. The Kruskal–Wallis Test was used to compare the amplitudes and latencies of the cortical components across these groups. For further analysis after the initial tests, the Dunn test was applied in the post-hoc phase.

## 3. Results

**Figure 2:**
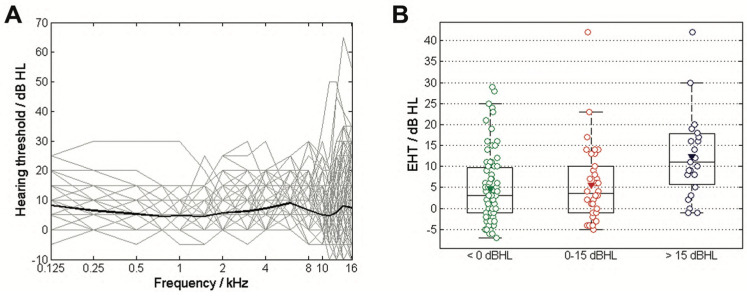
The hearing thresholds from 0.125 to 16 kHz for all participants are shown as gray lines, with the average threshold presented by a dark line (**A**). The boxplot (**B**) illustrates the average hearing threshold in the extended high-frequency range (EHT), categorized into three age groups: 20–25 years, 25–30 years, and 30–35 years. The mean values are indicated by triangles.

A total of seventy-three participants with normal hearing status were recruited in this study: 33 females and 40 males, with an average age of 25.8 ± 3.6 years, ranging from 21 to 35 years old. All 146 ears had normal hearing thresholds, with average hearing thresholds levels at 0.25, 0.5, 1, 2, and 4 kHz recorded at 5.5 ± 3.8 dB HL (Figure 2A). The hearing threshold at frequencies ranging from 0.125 to 8 kHz did not exceed 30 dB HL and was recorded at 6.1 ± 3.5 dB HL. Fourteen ears were excluded from further analysis because they did not show a measurable MMN response in at least one of the two stimulation paradigms (among them are four ears from two female participants, while the remaining ten excluded ears comprised four right ears and six left ears from ten different participants).

### 3.1. Age and EHT

In the selected ears, the average EHT at 10, 11.2, 12.5, 14, and 16 kHz was 6.5 ± 9.4 dB HL, with values ranging from −7 to 42 dB HL. After applying the exclusion criteria, the mean age of the participants whose ears were analyzed was 25.3 ± 3.5 years, with an age range of 21 to 35 years. The demographic distribution of ears according to age and EHT is summarized in Table 1. The EHT of three groups differed significantly (χ^2^ = 11.9, *p* = 0.03) as seen in Figure 2B. The post-hoc analysis revealed that the EHT for the 30–35-year age group was significantly higher than for those of the other two groups (*p* < 0.02, Cohen’s d = 0.88).

### 3.2. Cortical Response of the MMN Paradigms

We conducted two mismatch negativity (MMN) paradigms, each employing different acoustic stimuli: tonal and speech. After artifact reduction, the average number of recorded epochs was 165 for the standard stimulus (range: 50 to 194) and 43 for the deviant stimulus (range: 13 to 67). This corresponds to the exclusion of 17% of epochs for the standard stimulus and 32% for the deviant stimulus due to artifacts.

**Figure 3:**
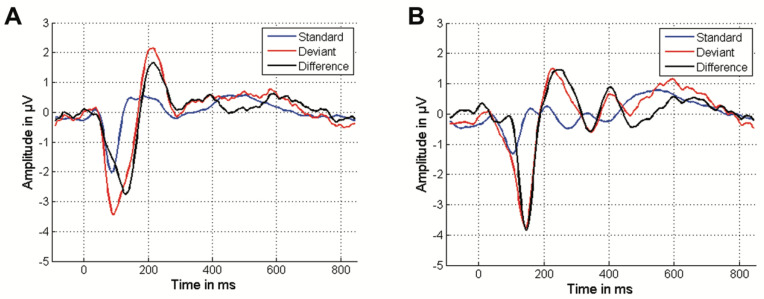
The grand average response to the standard stimulus (blue), the deviant stimulus (red), and the difference between the two stimuli (black) in the tone paradigm (**A**) and the speech paradigm (**B**). Both the standard and deviant stimuli elicit similar cortical reactions, characterized by pronounced negative N1 and positive P2 deflections. However, the response to the deviant stimulus is marked by a substantial negative deflection known as Mismatch Negativity (MMN), which is identifiable by examining the difference between the standard and deviant responses. The cortical response to the speech stimuli exhibits delayed latencies for N1, P2, and MMN compared to the tone stimulus paradigm. Additionally, a P300 component is elicited in the speech paradigm.

The responses to the standard stimulus, the deviant stimulus, and the difference between them are depicted as the grand average response for both conditions (Figure 3A,B).

In the tone paradigm (Figure 3A), two distinct potentials in the cortical response to the standard stimulus were identified (Figure 3A, blue). The first was a pronounced negative deflection with an amplitude of 2.01 µV occurring at a latency of 88 ms, associated with the N1 component. This was followed by a pronounced positive deflection, observed at a latency of 160 ms with an amplitude of 0.1 µV, corresponding to the P2 component. Considering the difference between the cortical response to the deviant and standard stimuli (Figure 3A, black), the MMN was successfully identified as a pronounced negative deflection with an amplitude of 2.75 µV at a latency of 130 ms.

In the speech paradigm (Figure 3B), the cortical response to the standard stimulus (Figure 3B, blue) displayed a pronounced negative deflection with an amplitude of 1.32 µV at a latency of 104 ms, indicative of the N1 component. This was followed by a double-peaked positive deflection reaching a maximum amplitude of 0.25 µV at a latency of 210 ms, associated with the P2 component. Regarding the difference of the cortical responses (Figure 3B, black), a distinct MMN was noted with an amplitude of 3.84 µV at 146 ms, accompanied by a pronounced positive deflection of 0.88 µV at a latency of 404 ms. This latter positive potential is attributed to the P300 component, reflecting attentional processes.

### 3.3. Comparison of the MMN Responses Grouped by Age and EHT

**Figure 4:**
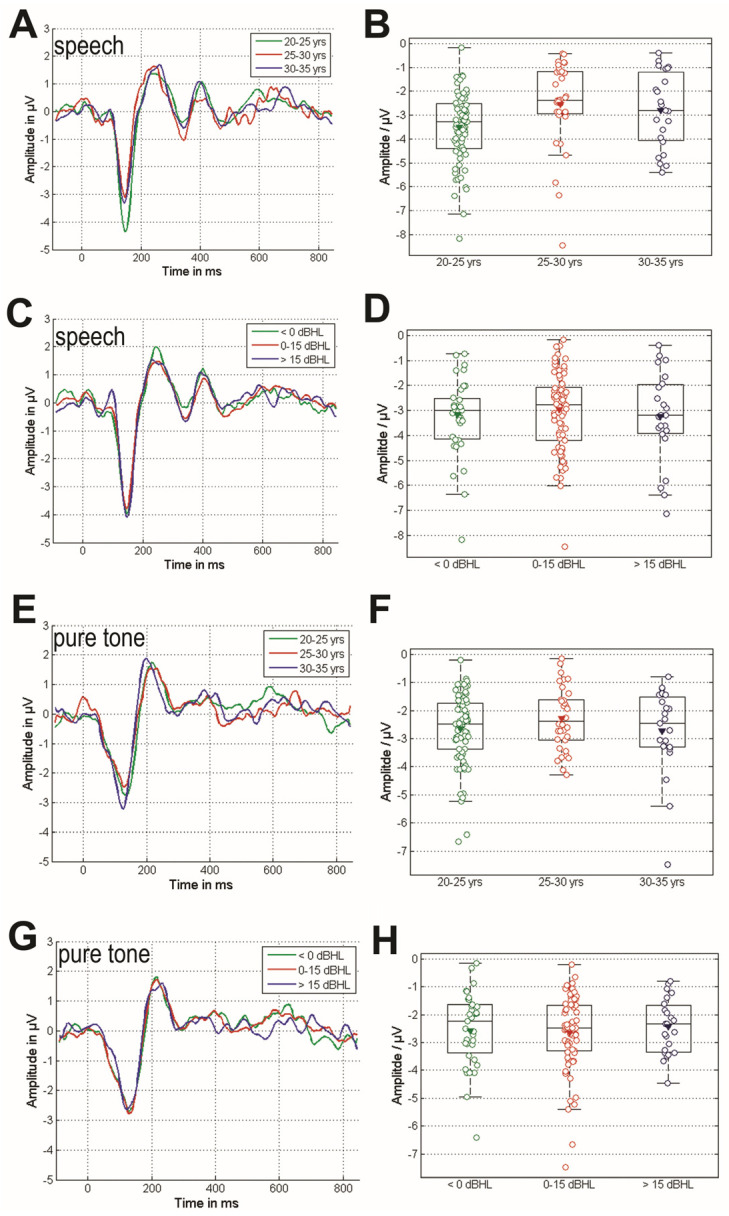
Comparison of cortical responses to difference between deviant and standard stimuli in speech (**A**–**D**) and tone (**E**–**H**) conditions. (**A**) Averaged cortical responses to speech stimuli across groups, categorized by participant ages: 20–25, 25–30, and 30–35 years old. (**B**) Age-based comparison of MMN amplitudes for grouped speech response data. (**C**) Averaged cortical responses to speech stimuli across groups, categorized by participants’ EHT: <0 dB HL, 0–15 dB HL, >15 dB HL and (**D**) corresponding MMN amplitude. (**E**) Averaged cortical responses to tone stimuli across groups, categorized by age and (**F**) corresponding MMN amplitude. (**G**) Averaged cortical responses to tone stimuli across groups, categorized by participants’ EHT and (**H**) corresponding MMN amplitudes. The mean values are indicated by triangles in all boxplots.

We found no significant correlation between MMN responses of the left and right ear, MMN responses and age, or MMN responses and EHT. However, significant effects were found when participants were grouped based on the effect being investigated. Figure 4 (on the left) displays the grand averages of these groups for both effects in the different responses across the two MMN paradigms (Speech: A–D, Tone: E–H); on the right side, the amplitudes per subject are compared using a boxplot. The comprehensive analysis encompassed not only the comparison of MMN amplitude but also the evaluation of MMN peak latency and the analysis of both amplitude and latency for the P300 potential within the speech paradigm (refer to Table 2). A significant age-related effect on the MMN amplitude in the speech condition was found (χ² = 13.0, *p* = 0.002)m with mean amplitudes and corresponding confidence intervals of A_20-25_ = −3.51 ± 0.33 µV, A_25-30_ = −2.54 ± 0.60 µV, and A_30-35_ = −2.8 ± 0.65 µV in each group. The post-hoc analysis showed a significant difference between the 20–25 yrs and 25–30 yrs groups (*p* = 0.0015, Cohen’s d = 0.63). Apart from that, no further effects of age were observed on the cortical potentials examined. Also, no significant influence of EHT on the amplitude or latency of the MMN or the P300 was detected for either the tone or speech paradigms.

## 4. Discussion

### 4.1. Summary of Findings of EHT and Cortical Potentials in Response to Tone and Speech Stimuli

Direct measurement of early changes in the peripheral auditory pathways, particularly cochlear synaptopathy, requires invasive methods that are not feasible in humans. As a non-invasive alternative, EHT has been considered a biomarker for early changes in the peripheral auditory pathway and may serve as an indirect marker for cochlear synaptopathy in humans [16,18]. This study found that individuals over 30 exhibited significantly higher EHT compared to younger groups, suggesting that early changes in the peripheral auditory pathway, potentially including cochlear synaptopathy, may already be present in these individuals. This finding aligns with previous research [18].

The aim of this study was to examine individual differences in central auditory processing in relation to age and EHT. The MMN, which is a marker of auditory discrimination ability, was chosen as a key measure since it is elicited even by small differences between two stimuli. To achieve this, oddball paradigms consisting of a standard stimulus and a deviant were employed, using two different stimulus pairs: a tone-based paradigm (1 kHz and 2 kHz) and a speech-based paradigm (“ba” and “da”). In both paradigms, the MMN component was successfully extracted by calculating the difference between cortical responses to the standard and deviant stimuli (Figure 3).

Notably, in both paradigms, the MMN amplitude was larger than the N1 amplitude for both deviant and standard stimuli. This may be attributed to the electrode placement at Fpz, where MMN amplitudes tend to be higher [37], whereas the N1–P2 complex typically exhibits higher amplitudes when recorded at Cz [38].

In the tone paradigm, it was unexpectedly found that subtracting the standard stimulus did not fully eliminate the N1–P2 complex of the deviant stimulus. The reason for this incomplete regression remains unclear but may be related to the properties of the stimuli and not with the recording setup, as both were recorded with the same electrode positions. The N1–P2 complex is known to be sensitive to loudness effects [39]. However, it is unlikely that differences in perceived loudness contributed to this effect, as both stimuli were calibrated to 70 dB nHL, and participants’ hearing thresholds at 1 kHz and 2 kHz showed only minimal variation. A more plausible explanation are differences in the frequency dependent interstimulus interval (ISI) between the deviant and standard stimulus. Longer ISI has been shown to specifically reduce P2 component amplitudes due to loudness adaptation [40]. For the N1 component, this regression approach is considered a robust method for isolating the MMN component [41]. In our data, this approach successfully regressed out the N1 component in the speech paradigm, as expected. Although this issue does not significantly affect the interpretation of our results—since the regression artifact equally impacts all groups—further data collection would be valuable to explore this effect in greater detail. Future studies utilizing EEG recordings with multiple channels, which enable higher spatial resolution analyses and the possibility to localize dipole sources in the brain, along with additional cortical responses for each stimulus, would allow for a more thorough examination of this aspect.

Furthermore, a distinct endogenous P300 potential was successfully recorded in the speech paradigm without the need for an additional task, whereas it was absent in the tone paradigm. This unexpected finding contrasts with previous studies [42,43] and may be due to the substantial spectral and temporal differences between the stimuli “da” and “ba” used in this study, which could have involuntarily captured the participants’ attention [44].

### 4.2. The Relation of MMN and EHT

When participants were classified into different EHT-based groups and compared in terms of MMN responses, no significant differences were found in amplitude or latency for either pure-tone or speech stimuli. However, previous studies suggest that high-frequency audiometric thresholds can influence cortical auditory evoked potentials [45,46,47], including the MMN. For example, Chen et al. (2016) showed that increasing high-frequency hearing thresholds correlate with prolonged speech-evoked MMN latency in noisy environments [45]. Zeng et al. (2020) found that hearing loss across the frequency range between 125 Hz and 8 kHz impairs suprathreshold processing and interferes with speech perception [47]. We believe that the differences in results may be attributed to variations in hearing threshold deterioration across different frequency ranges. In this study, the focus was set on EHT in a frequency range from 10 to 16 kHz, while the other study examined frequencies from 4 to 8 kHz. As we know, the stimulus of ‘da/ba’ is primarily located within frequencies up to around 4 kHz [48]. Therefore, differencing between these stimuli may not engage a central processing area above 8 kHz.

As mentioned previously, animal studies have shown that OHCs in the basal turn exhibit lower sensitivity compared to cochlear synapses in the apical and middle turns. In animals, OHC loss at the basal turn, particularly in the high-frequency range, is strongly associated with cochlear synaptopathy in the apical and middle turns. Consequently, high-frequency threshold deterioration in animals can serve as an indirect indicator of cochlear synaptopathy [23,24]. While animal data cannot be directly extrapolated to humans, these findings suggest that EHT elevation, as an early sign of peripheral auditory pathway changes, could similarly serve as an indirect biomarker for cochlear synaptopathy, particularly in the apical and middle turns of the cochlea in humans. Based on these assumptions, our findings suggest that the early changes in the peripheral auditory pathway, including potential cochlear synaptopathy in the apical and middle turns, may not significantly impact central auditory processing. However, a major limitation of this study is the absence of a direct approach to verify early auditory pathway changes in the participants histologically, including potential cochlear synaptopathy.

### 4.3. The Relation Between MMN and Aging

Previous studies have reported that aging not only reduces the amplitude of the MMN response but also prolongs its latency [49,50,51,52,53,54]. However, the work by Kisley et al. (2005) compared only older adults (over 55 years old) with young individuals aged 18–23 years old [52]. Since aging is often associated with high-frequency hearing loss, it is unclear whether the observed MMN effect is due to age-related changes or high-frequency hearing loss. The study’s criteria only considered hearing thresholds at 1 kHz with a limit of 10 dB HL, without accounting for other frequencies. In another study by Ruzzoli et al. (2012), MMN was compared across three age groups: (1) 21–40 years, (2) 41–60 years, and (3) 61–80 years, revealing that MMN was diminished in the oldest group but showing no difference between the young and middle-aged groups [53]. However, the study only briefly mentioned conducting a hearing test to exclude sensorineural hearing loss, without specifying the test frequencies or criteria. In contrast, the present study enrolled young adults aged 21–35 years with normal hearing thresholds from 0.125 kHz to 8 kHz only. Here, MMN amplitudes decrease with age.

Another aspect is that most previous studies used pure tones as stimuli and observed aged-related changes in MMN [52,53]. Pure-tone and speech stimuli applied to all the participants in this study led to the observation of age-related changes only in response to speech stimuli. This finding suggests that early changes in the central nervous system during aging, similar to hidden hearing loss in the peripheral auditory system, may affect complex listening situations or the capacity to understand complex speech stimuli, while leaving simple tone differentiation unaffected [55,56].

Finally, this study found no age-related effect on MMN latency in response to either speech or pure-tone stimuli. This suggests that the speed of signal propagation in the nervous system may remain largely unaffected in the early phase of aging, indicating that significant demyelination in the central auditory system has not yet occurred. This indicates that MMN latency is a less sensitive and appropriate biomarker for evaluating central speech recognition. Similar findings have been reported in studies involving cochlear implant patients [37].

## 5. Limitations

The measurement of MMN in this study has several limitations. Firstly, we used only three active electrodes, which limits the ability to differentiate distinct MMN generators. Secondly, the differentiation between standard and deviant stimuli is imprecise when using speech stimuli. Specifically, the ‘da’ deviant response is a superposition of the ‘da’ cortical response and the MMN. Regarding the ‘da’ cortical response, the paradigm must be repeated with ‘da’ as the standard stimulus. However, this was not implemented in the present study due to time constraints. Moreover, there is currently no direct evidence of cochlear synaptopathy in humans. The possibility of pathological changes in the peripheral and central nervous systems was not directly observed or confirmed in the participants. Additionally, the age range in this study was limited to only 20–35 years old. Future studies recruiting a broader age range may reveal clearer age-related effects. Furthermore, we did not use a professional questionnaire to assess noise exposure; instead, we asked participants to actively recall their history of noise exposure. This method may introduce bias and does not fully eliminate the potential effects of noise exposure. Moreover, a limited number of repetitions were conducted for both the standard stimulus and, in particular, the deviant stimulus, which may have increased variability in amplitude and introduced bias in the conclusions. The resulting differences in the signal-to-noise ratio between subjects can lead to smearing the EEG signal when averaging across participants. This limitation will need to be addressed in future studies.

## 6. Conclusions

During the early stages of age-related hearing loss, pathological changes occur in the peripheral and central auditory systems. EHT was used as an indirect biomarker to detect pathology, potentially associated with cochlear synaptopathy in the peripheral auditory system; however, it is not suitable for detecting changes in the central auditory system. The application of using MMN as an electrophysiological tool to diagnose hidden hearing loss in normal hearing adults has limitations. However, in contrast to MMN responses to tonal stimuli, the present study reveals that MMN amplitudes obtained with speech stimuli may indicate early signs of central auditory deficits.

## Figures and Tables

**Table 1:** Demographic table for participants. EHT for each group is presented as mean and standard deviation.

Group		20–25 Years	25–30 Years	30–35 Years	Total
Number of subjects		40	19	12	71
Number of ears		75	34	23	132
Sex	M	43	16	11	72
	F	32	18	12	62
EHT in dB HL		4.8 ± 8.4	5.6 ± 9.6	12.4 ± 10.1	6.5 ± 9.4

**Table 2:** Kruskal–Wallis test to analyze the relationship between age and EHT and electrophysiological metrics: Amplitude and Latency of MMN and P300.

Categorical Group	Metric	χ²_Amplitude_	P_Amplitude_	χ²_Latency_	P_Latency_
Age	MMN_Tone_	1.29	0.53	4.64	0.10
	MMN_Speech_	13.0	0.002	1.87	0.39
	P300_Speech_	4.24	0.12	0.19	0.91
EHT	MMN_Tone_	0.12	0.94	0.94	0.62
	MMN_Speech_	0.69	0.71	2.86	0.24
	P300_Speech_	0.21	0.90	0.96	0.62

## Data Availability

The data that support the findings of this study are available from the corresponding author, (L.Z.) upon reasonable request.

## Abbreviations

The following abbreviations are used in this manuscript:

ARHL	age-related hearing loss
EFR	envelop-following response
EHT	extended high-frequency threshold
MEMR	acoustic middle ear muscle reflex
MMN	mismatch negativity
OHC	outer hair cell

## References

[1] Agrawal Y., Platz E.A., Niparko J.K. (2008). Prevalence of hearing loss and differences by demographic characteristics among US adultsdata from the national health and nutrition examination survey, 1999–2004. Arch. Intern. Med..

[2] Yamasoba T., Lin F.R., Someya S., Kashio A., Sakamoto T., Kondo K. (2013). Current concepts in age-related hearing loss: Epidemiology and mechanistic pathways. Hear. Res..

[3] (2018). Addressing the Rising Prevalence of Hearing Loss.

[4] Wang J., Puel J.-L. (2020). Presbycusis: An update on cochlear mechanisms and therapies. J. Clin. Med..

[5] Basner M., Babisch W., Davis A., Brink M., Clark C., Janssen S., Stansfeld S. (2014). Auditory and non-auditory effects of noise on health. Lancet.

[6] Liberman M.C., Kujawa S.G. (2017). Cochlear synaptopathy in acquired sensorineural hearing loss: Manifestations and mechanisms. Hear. Res..

[7] Long P., Wan G., Roberts M.T., Corfas G. (2018). Myelin development, plasticity, and pathology in the auditory system. Dev. Neurobiol..

[8] Mulders W.H., Chin I.L., Robertson D. (2018). Persistent hair cell malfunction contributes to hidden hearing loss. Hear. Res..

[9] Schaette R., McAlpine D. (2011). Tinnitus with a normal audiogram: Physiological evidence for hidden hearing loss and computational model. J. Neurosci. Off. J. Soc. Nurosci..

[10] Sergeyenko Y., Lall K., Liberman M.C., Kujawa S.G. (2013). Age-related cochlear synaptopathy: An early-onset contributor to auditory functional decline. J. Neurosci..

[11] Moser T., Predoehl F., Starr A. (2013). Review of hair cell synapse defects in sensorineural hearing impairment. Otol. Neurotol..

[12] Kujawa S.G., Liberman M.C. (2009). Adding insult to injury: Cochlear nerve degeneration after “temporary” noise-induced hearing loss. J. Neurosci..

[13] Kujawa S.G., Liberman M.C. (2015). Synaptopathy in the noise-exposed and aging cochlea: Primary neural degeneration in acquired sensorineural hearing loss. Hear. Res..

[14] Shaheen L.A., Valero M.D., Liberman M.C. (2015). Towards a diagnosis of cochlear neuropathy with envelope following responses. J. Assoc. Res. Otolaryngol..

[15] Bramhall N., Beach E.F., Epp B., Le Prell C.G., Lopez-Poveda E.A., Plack C.J., Schaette R., Verlust S., Canlon B. (2019). The search for noise-induced cochlear synaptopathy in humans: Mission impossible?. Hear. Res..

[16] Liberman M.C., Epstein M.J., Cleveland S.S., Wang H., Maison S.F. (2016). Toward a differential diagnosis of hidden hearing loss in humans. PLoS ONE.

[17] Bramhall N.F. (2021). Use of the auditory brainstem response for assessment of cochlear synaptopathy in humans. J. Acoust. Soc. Am..

[18] Schmidt F.H., Dörmann A., Ehrt K., Grossmann W., Mlynski R., Zhang L. (2024). The curvature quantification of wave I in auditory brainstem responses detects cochlear synaptopathy in human beings. Eur. Arch. Otorhnilaryngol..

[19] James J.A., Park J.H., Smith E.M., Johnson C.E., Clifton S., Danhauer J.L., Barbee C.M. (2018). Effectiveness of auditory measures for detecting hidden hearing loss and/or cochlear synaptopathy: A systematic review. Semin. Hear..

[20] Pappa A.K., Hutson K.A., Scott W.C., Wilson J.D., Fox K.E., Masood M.M., Giardina C.K., Pulver S.H., Grana G.D., Askew C. (2019). Hair cell and Neural contributions to the cochlear summating potential. J. Neurophysiol..

[21] Yaşar M., Öner F., Atalay F., Anbar S.S. (2025). Cochlear synaptopathy evaluation with Electrocochleography in patients with hearing difficulty in noise despite normal hearing levels. Clin. Otolaryngol..

[22] Haggerty R.A., Hutson K.A., Riggs W.J., Brown K.D., Pillsbury H.C., Adunka O.F., Buchman C.A., Fitzpatrick D.C. (2023). Assessment of cochlear synaptopathy by electrocochleography to low frequencies in a preclinical model and human subjects. Front. Neurol..

[23] Maison S.F., Usubuchi H., Liberman M.C. (2013). Efferent feedback minimizes cochlear neuropathy from moderate noise exposure. J. Neurosci..

[24] Liberman M.C., Liberman L.D., Maison S.F. (2014). Efferent feedback slows cochlear aging. J. Neurosci..

[25] Chambers A.R., Resnik J., Yuan Y., Whitton J.P., Edge A.S., Liberman M.C., Polley D.B. (2016). Central gain restores auditory processing following near-complete cochlear denervation. Neuron.

[26] Gu J.W., Halpin C.F., Nam E.-C., Levine R.A., Melcher J.R. (2010). Tinnitus, diminished sound-level tolerance, and elevated auditory activity in humans with clinically normal hearing sensitivity. J. Neurophysiol..

[27] Sun W., Deng A., Jayaram A., Gibson B. (2012). Noise exposure enhances auditory cortex responses related to hyperacusis behavior. Brain Res..

[28] Chen G.-D., Radziwon K.E., Kashanian N., Manohar S., Salvi R. (2014). Salicylate-induced auditory perceptual disorders and plastic changes in nonclassical auditory centers in rats. Neural. Plast..

[29] Noreña A.J. (2011). An integrative model of tinnitus based on a central gain controlling neural sensitivity. Neurosci. Biobehav. Rev..

[30] Auerbach B.D., Rodrigues P.V., Salvi R.J. (2014). Central gain control in tinnitus and hyperacusis. Front. Neurol..

[31] Näätänen R., Gaillard A., Mäntysalo S. (1978). Early selective-attention effect on evoked potential reinterpreted. Acta Psychol..

[32] Näätänen R. (1995). The mismatch negativity: A powerful tool for cognitive neuroscience. Ear Hear..

[33] Näätänen R., Petersen B., Torppa R., Lonka E., Vuust P. (2017). The MMN as a viable and objective marker of auditory development in CI users. Hear. Res..

[34] Lonka E., Kujala T., Lehtokoski A., Johansson R., Rimmanen S., Alho K., Näätänen R. (2004). Mismatch negativity brain response as an index of speech perception recovery in cochlear-implant recipients. Audiol. Neurotol..

[35] British Society of Audiology Recommended Procedure: Pure Tone Air and Bone Conduction Threshold Audiometry with and Without Masking and Determination of Uncomfortable Loudness Levels. 2004. https://www.thebsa.org.uk/.

[36] Riedel H., Granzow M., Kollmeier (2001). Single-sweep-based methods to improve the quality of auditory brain stem responses Part II: Averaging methods. Z. Audiol..

[37] Turgeon C., Lazzouni L., Lepore F., Ellemberg D. (2014). An objective auditory measure to assess speech recognition in adult cochlear implant users. Clin. Neurophysiol..

[38] Näätänen R., Picton T. (1987). The N1 Wave of the human electric and magnetic response to sound: A review and an analysis of the component structure. Psychophysiology.

[39] Schmidt F.H., Mauermann M., Kollmeier B. (2020). Neural representation of loudness: Cortical evoked potentials in an induced loudness reduction experiment. Trends Hear..

[40] Lanting C.P., Briley P.M., Sumner C.J., Krumbholz K. (2013). Mechanisms of adaption in human auditory cortex. J. Neurophysiol..

[41] Näätänen R., Paavilainen P., Rinne T., Alho K. (2007). The mismatch negativity (MMN) in basic research of central audiotry processing: A review. Clin. Neurophyiol..

[42] Tao D., Zhang Y., Liu H., Zhang W., Xu M., Galvin J.J., Zhang D., Liu J. (2022). The P300 auditory event-related potential may predict segregation of competing speech by bimodal cochlear implant listeners. Front. Neurosci..

[43] Polich J. (2007). Updating P300: An integrative theory of P3a and P3b. Clin. Neurophysiol..

[44] Tervaniemi M., Lehtokoski A., Sinkkonen J., Virtanen J., Ilmoniemi R., Näätänen R. (1999). Test–retest reliability of mismatch negativity for duration, frequency and intensity changes. Clin. Neurophysiol..

[45] Chen J., Chen S., Zheng Y., Ou Y. (2016). The effect of aging and the high-frequency auditory threshold on speech-evoked mismatch negativity in a nosiy background. Audiol. Neurootol..

[46] Chen J., Zhao Y., Zou T., Wen X., Zhou X., Yu Y., Liu Z., Li M. (2022). Sensorineural hearing loss affects functional connectivity of auditory cortex, parahipocampal gyrus and inferior prefrontal gyrus in tinnitus patients. Front. Neurosci..

[47] Zeng F.-G., Richardson M., Turner K. (2020). Tinnitus does not interfere with auditory and speech perception. J. Neurosci..

[48] Berthommier F., Girin L., Boe L.J. A simple hybrid acoustic/morphologically constrained technique for the synthesis of stop consonants in various vocalic contexts. Proceedings of the Interspeech 2012—13th Annual Conference of the International Speech Communication Association.

[49] Pekkonen E., Jousmäki V., Partanen J., Karhu J. (1993). Mismatch negativity area and age-related auditory memory. Electroencephalogr. Clin. Neurophysiol..

[50] Pekkonen E., Rinne T., Reinikainen K., Kujala T., Alho K., Naatanen R. (1996). Aging effects on auditory processing: An event-related potential study. Exp. Aging Res..

[51] Cooper R.J., Todd J., McGill K., Michie P.T. (2006). Auditory sensory memory and the aging brain: A mismatch negativity study. Neurobiol. Aging.

[52] Kisley M.A., Davalos D.B., Engleman L.L., Guinther P.M., Davis H.P. (2005). Age-related change in neural processing of time-dependent stimulus features. Cogn. Brain Res..

[53] Ruzzoli M., Pirulli C., Brignani D., Maioli C., Miniussi C. (2012). Sensory memory during physiological aging indexed by mismatch negativity (MMN). Neurobiol. Aging.

[54] Näätänen R., Kujala T., Escera C., Baldeweg T., Kreegipuu K., Carlson S., Ponton C. (2012). The mismatch negativity (MMN)—A unique window to disturbed central auditory processing in aging and different clinical conditions. Clin. Neurophysiol..

[55] Maddox J. (1994). Cocktail party effect made tolerable. Nature.

[56] Starr A., Sininger Y., Pratt H. (2000). The varieties of auditory neuropathy. J. Basic Clin. Physiol. Pharmacol..

